# Effect of septoplasty on left ventricular myocardial performance in patients with nasal septum deviation

**DOI:** 10.1016/j.bjorl.2020.08.004

**Published:** 2020-09-26

**Authors:** Hakan Kaya, Ercan Kurt, Mehtap Koparal, Hakan Tibilli, Yusuf Hosoglu, Safiye Kafadar, Arif Suner, Serdar Türkmen

**Affiliations:** a Adiyaman University, Faculty of Medicine, Department of Cardiology, Adiyaman, Turkey; b Adiyaman Educational and Resarch Hospital, Department of Otorhinolarygology, Adiyaman, Turkey; c Adiyaman Educational and Resarch Hospital, Department of Cardiology, Adiyaman, Turkey; d Adiyaman University, Faculty of Medicine, Department of Radiology, Adiyaman, Turkey

**Keywords:** Echocardiography, Myocardial performance index, Nasal septum deviation, Septoplasty

## Abstract

**Introduction:**

Nasal septum deviation is the leading cause of upper airway obstruction. Chronic upper airway obstruction may cause myocardial injury due to chronic hypoxia. Effects of septoplasty on left venticular diastolic and sistolic functions are not well known. The myocardial performance index is an easy-to-apply and reliable parameter that reflects systolic and diastolic cardiac functions.

**Objective:**

The present study aimed to investigate the effect of nasal septoplasty on the myocardial performance index in patients with nasal septal deviation.

**Methods:**

This prospective study consisted of 50 consecutive patients who underwent septoplasty due to symptomatic prominent C- or S-shaped nasal septal deviation. Transthoracic echocardiogarphy was performed in all patients before and 3 months after septoplasty. Calculated myocardial performance indices were compared.

**Results:**

Significantly higher left ventricular myocardial performance index (0.52 ± 0.06 vs. 0.41 ± 0.04, *p* <  0.001), longer isovolumic relaxation time (95.0 ± 12.5 vs. 78.0 ± 8.6 ms, *p* <  0.001), longer isovolumic contraction time (45.5 ± 7.8 vs. 39.5 ± 8.6 ms, *p* <  0.001), longer deceleration time (184.3 ± 32.5 vs. 163.6 ± 45.4 ms, *p* =  0.004), higher ratio of transmitral early to late peak velocities (E/A) (1.42 ± 0.4 vs. 1.16 ± 0.2, *p* =  0.006) and shorter ejection time (270.1 ± 18.3 vs. 286.5 ± 25.8 ms, *p* <  0.001) were observed before septoplasty when compared to values obtained 3 months after septoplasty. Left ventricular systolic ejection fraction was similar before and after septoplasty (63.8±2.8% vs. 64.6±3.2%, *p*  = 0.224).

**Conclusion:**

Septoplasty surgery not only reduces nasal blockage symptoms in nasal septal deviation patients but also may improve left ventricular performance. Thus, treatment of nasal septal deviation without delay is suggested to prevent possible future cardiovascular events.

## Introduction

There are various pathologies leading to nasal obstruction such as concha bullosa, inferior nasal concha hypertrophy and nasal polyposis; however, nasal septal deviation (NSD) still remains the main ethiologic cause.^1^, ^2^ These deformities all cause decrease in nasal airflow; thus, autonomic dysfunction due to induced hypoxia and hypercapnia triggers cardiac arrhythmias; septoplasty is shown to reduce these arrhytmias as a consequence.^3^

Chronic hypoxia and hypercapnia can cause myocardial injury in patients with NSD. Myocardial injury can cause heart failure due to diastolic dysfunction without systolic dysfunction. Heart failure is due not only to left ventricular systolic dysfunction, but also to left ventricular diastolic dysfunction. There are few Doppler echocardiographic variables that combine left ventricular systolic and diastolic measurements. Two-dimensional echocardiography and Doppler echocardiography provide important information about systolic and diastolic functions of the left ventricle. The myocardial performance index (MPI) is easily reproducible and reliable echocardiographic parameter reflecting both diastolic and systolic myocardial functions.^4^, ^5^, ^6^ Echocardiography has been recently used to assess cardiac functions in patients with upper airway obstruction. However, only pulmonary artery pressure and right ventricle functios are evaluated in the literature. Effects of hypoxia and hypercapnia caused by NSD on left ventricular systolic and diastolic functions have not yet been fully studied. In the present study, we aimed to asses MPI of left ventricle in patients with NSD and investigate the effects of septoplasty on MPI in this population.

## Methods

### Study population and design

This prospective study was conducted in accordance with the Declaration of Helsinki and aproved by the local Ethics Commitee. Patients with symptomatic and definite NSD are included in the study population. Persistent nasal obstruction related to septal deviation despite the optimal medical treatment for at least 4 weeks was determined as the indication for septoplasty. Optimal medical treatment includes topical nasal steroids, topical or oral decongestants, or an oral antihistamine/decongestant combination. Nasal endoscopy, paranasal sinus computed tomography, fiberoptic nasal and nasopharengeal exam were performed to exclude additional nasal abnormalities causing upper airway obstruction. Patients with sinusitis, allergic rhinitis, cocha bullosa and obstructive inferior nasal concha hypertrophy, more than 50% velopharygeal and hypopharyngeal obstruction and Grade 3 − 4 tonsil hypertrophy according to Brodsky scala were excluded. Patients with hypertension, heart failure, coronary artery disease, chronic pulmonary diseases, valvuler heart diseases, cardiac arrhythmias and conduction abnormalities and pacemakers were also excluded.

The study included 67 consecutive patients older than 18 years referred to the otolaryngology clinic who underwent septoplasty operation. 17 patients were excluded from the study according to exclusion criteria. Finally, the study consisted of 50 patients. Complete ear, nose and throat examination was performed on all participants. Echocardiography and Nasal Obstruction Septoplasty Effectiveness (NOSE) scale were performed on all patients before and 3 months after the septoplasty.

### NOSE scale

The NOSE scale consists of 5 subsets of a questionnaire: i) nasal congestion, ii) partial or complete nasal obstruction, iii) difficulty in breathing through the nose, iv) trouble sleeping, and v) difficulty getting adequate air through the nose on exertion, which requires a rating from 0 to 4, ranging from no problem to a severe problem that corresponds to minimum value of 0 and maximal value of 20. Acquired scores from the NOSE scale are recommended to be multiplied by 5, because the quality of life scales are usually given between 0 and 100 percent. The Dreher classification was used to evaluate the degree of nasal septal deviation (0: no deviation, 1: mild deviation, 2: moderate deviation and 3: severe deviation).^7^

### Surgical technique

A hemitransfixion or Killian incision was performed in all patients under general anesthesia. Mucoperichondrial and mucoperiosteal flaps were elevated and reflected, and then the cartilage and bony structures were removed, reshaped, and replaced in proper position on the both sides of the nasal septum. Intranasal septal splints with airway were applied following transseptal suturing. Finally, we removed the splints 1 week after the surgery. Septoplasty operations were done by experienced surgeons.

### Echocardiographic examination

**Figure 1:**
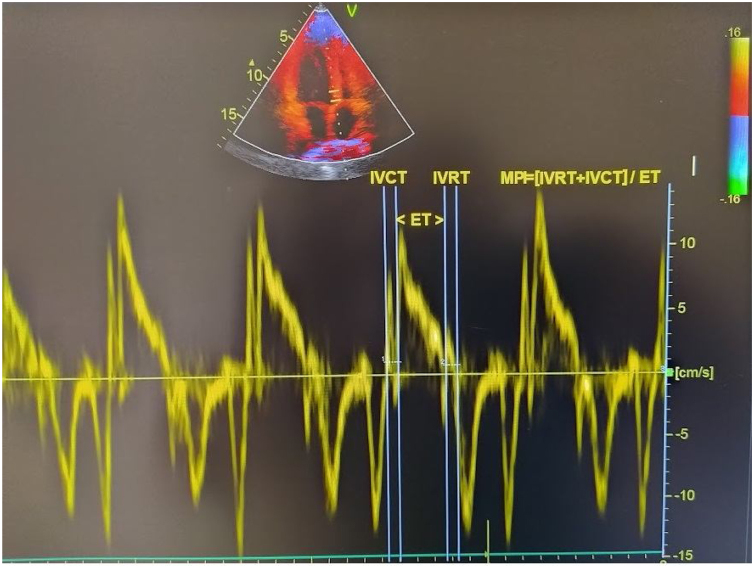
Tissue doppler-derived MPI.

Transthoracic echocardiographic examinations were performed to all patients before and 3 months after the septoplasty using a machine (Vivid 5S General Electric Medical System, Horten, Norway) with a 2.0–3.5 MHz transducer. Doppler and Two-dimensional echocardiography was done in the standard views according to the guidelines of the American Society of Echocardiography.^8^ The modified Simpson’s method was used to calculate the Left Ventricular Ejection Fraction (LVEF).^9^ Pulsed wave doppler recordings of the mitral inflow velocities are obtained from apical four-chamber views by placing the sample volume between the tips of the mitral leaflets and then the peak late (A) and early (E) transmitral filling velocities and E/A ratio were measured. Isovolumic Relaxation Time (IVRT) defined as the time between closing the aortic valve and opening the mitral valve, Isovolumic Contraction Time (IVCT) defined as the time between closing the mitral valve and opening the aortic valve, Ejection Time (ET) defined as the time between opening and closing of the aortic valve were measured by using pulse wave recordings obtained from the transducer located between LV out flow tract and mitral valve. MPI was calculated by using the equation: MPI=(IVCT + IVRT)/ET.^6^ Tissue Doppler measurements were obtained from an average of five consecutive pulsed wave tissue doppler imaging performed from an apical four-chamber view with a sample volume of 2 mm placed on the lateral wall of the mitral annulus. Echocardiographic measurements were made by experienced cadiologists. The calculation of tissue doppler-derived MPI is presented in Fig. 1.

### Statistical analysis

The SPSS, v. 22.0, statistical progam (Chicago, USA) was used for the statistical analyses. All values are given as mean ± standard deviation. Independent *t*-test or Mann-Whitney *U* Test was performed on each group to compare the numerical variables. Distribution of variables are assessed by Kolmogorov-Smirnov test. The categorical variables were compared with Chi-square test; p-value of < 0.05 was considered statistically significant.

## Results

Clinical characteristics and echocardiogarphic findings of the patients are presented in Table 1. Mean age was 32.6 ± 10.5 and 64% were male. The preoperative NOSE scale was observed significantly higher when compared to after septoplasty (68 ± 10.6 vs. 30.5 ± 10.1, *p* < 0.001) Significantly higher left ventricular MPI (0.52 ± 0.06 vs. 0.41 ± 0.04, *p* <  0.001), longer IVRT (95.0 ± 12.5 vs. 78.0 ± 8.6 ms, *p* <  0.001), longer IVCT (45.5±7.8 vs. 39.5±8.6 ms, *p* <  0.001), longer deceleration time (184.3 ± 32.5 vs. 163.6 ± 45.4 ms, *p* =  0.004), higher E/A ratio (1.42 ± 0.4 vs. 1.16 ± 0.2, *p* =  0.006) and shorter ET (270.1 ± 18.3 vs. 286.5 ± 25.8 ms, *p* <  0.001) values were observed before septoplasty compared to the values 3 months after septoplasty. Left ventricular systolic ejection fractions and heart rates were similar before and after septoplasty (63.8 ± 2.8% vs. 64.6 ± 3.2%, *p* =  0.224, 76.8 ± 13 vs. 73.2 ± 12 beats/min, *p* =  0.546, respectively). There were no significant changes in BMI, diastolic and systolic blood pressure after surgery compared to basal values (23.5 ± 5.3 vs. 22.3 ± 4.8 kg/m^2^, *p* =  0.068, 122.5 ± 7.4 vs. 120.3 ± 5.2 mmHg, *p* =  0.614, 74.5 ± 4.8 vs. 73.7 ± 3.5 mmHg, *p* =  0.738, respectively).

**Table 1:** Clinical characteristics and echocardiogarphic findings of the patients.

	Before septoplasty	3 months after septoplasty	*p*-value
Age (years)	32.6 ± 10.5		
Gender, male (%)	32 (64)		
BMI (kg/m^2^)	23.5 ± 5.3	22.4 ± 4.9	0.068
Systolic BP (mmHg)	122.5 ± 7.4	120.3 ± 5.2	0.614
Diastolic BP (mmHg)	74.5 ± 4.8	73.7 ± 3.5	0.738
NOSE scale	68 ± 10.6	30.5 ± 10.1	<0.001
Heart rate (beats/min)	76.8 ± 13	73.2 ± 12	0.546
LVEF (%)	63.8 ± 2.8	64.6 ± 3.2	0.224
MPI	0.52 ± 0.06	0.41 ± 0.04	<0.001
IVRT (ms)	95.0 ± 12.5	78.0 ± 8.6	<0.001
IVCT (ms)	45.5 ± 7.8	39.5 ± 8.6	<0.001
E/A	1.42 ± 0.4	1.16 ± 0.2	0.006
ET (ms)	270.1 ± 18.3	286.5 ± 25.8	<0.001
DT (ms)	184.3 ± 32.5	163.6 ± 45.4	0.004

BMI, Body Mass Index; BP, Blood Pressure; NOSE, Nasal Obstruction Septoplasty effectiveness; LVEF, Left Ventricular Systolic Ejection Fraction; MPI, Myocardial Performance Index; IVRT, Isovolumic Relaxation Time; IVCT, Isovolumic Contraction Time; E/A, the ratio of transmitral early to late peak velocities; ET, Ejection Time; DT, Deceleration Time.

## Discussion

This study showed that MPI values decreased significantly 3 months after the septoplasty in patients with NSD. According to our findings left ventricular performance was subclinically impaired in patients with NSD and this impairment may be reversible after ungoing septoplasty. To our knowledge, this is the first study that evaluated MPI, including both systolic contraction and diastolic relaxation periods of the left ventricle, before and after septoplasty surgery. Nasal septal deviation is the most common cause of partial or complete chronic upper airway obstruction.^10^ Minor changes in the nasal patency could affect the total airway resistance, because nasal airflow resistance constitutes approximately half of the respiratory resistance.^10^, ^11^ Recently, there have been increased interest in cardiovascular complications of upper airway obstruction concerning obstructive sleep apnea syndrome, which sometimes might result in right heart failure, cor pulmonale, and even sudden death.^12^, ^13^ Most of patients with uncomplicated NSD would be asymptomatic on cardiological aspects; however, they may still be at risk for future cardiovascular diseases that would be encountered at older ages.

Effective septoplasty surgery provides improvement in scales of health-related quality of life not only because it corrects nasal airflow but also this rearrangement has positive systemic effects, especially on the cardiovascular system. Celiker et al. showed that septoplasty reduces sympathetic tonus which triggers cardiac arrhythmias by assessing heart rate variability in patients with NSD.^14^ Yurttas et al. showed negative effects of upper airway obstruction on cardiac arrhythmias and improvement after surgery by electrocardiography and Holter analyses in patients with NSD.^15^ One of the effects on cardiac functions concerning NSD is a nasocardiac reflex where the afferent arc of the reflex is represented by the maxillary branch of trigeminal nerve and the efferent arc is via the vagus nerve.^1^ Elicitation of this reflex is manifested as apnea, bradycardia or even asystole. A previous study showed that under general anesthesia stimulation of nasal mucosa with a nasal speculum had caused bradycardia via a nasocardiac reflex.^16^ Besides, NSD was demonstrated to cause chronic parasympathetic activity by permanent stimulation of this reflex arc.^1^

Echocardiography has been recently used to assess cardiac functions in patients with upper airway obstruction.^17^ However, only pulmonary artery pressure and right ventricle functions are evaluated in these studies.^18^, ^19^, ^20^ Effects of hypoxia and hypercapnia caused by NSD on left ventricular systolic and diastolic functions have not yet been fully studied. In our study, the left ventricular diastolic and systolic functions in patients with NSD were assessed using several echocardiographic parameters before and after septoplasty. Although systolic and diastolic dysfunctions appear together, there are limited doppler echocardiogram variables that combine systolic and diastolic measurements. We used the MPI which includes systolic contraction and diastolic relaxation periods of the left ventricle.^21^ MPI is a feasible and reliable method as it is not significantly affected by preload, afterload, sample volume location, age and heart rhythm.^22^, ^23^ The value of MPI in left ventricular dysfunction has been validated in the patients with idiopathic dilated cardiomyopathy and symptomatic heart failure with nonischemic or ischemic etiology.^24^, ^25^ In our study, MPI was shown to be higher in patients with NSD before septoplasty. However, it was demonstrated that MPI levels significantly decreased after septoplasty surgery. According to our findings, patients with NSD have subclinical left ventricular diastolic dysfunction although, LV systolic functions clinically seemed to be normal. This diastolic impairment may be reversible after septoplasty. The impairment in left ventricular performance in patients with NSD might be explained by hypoxia, hypercapnia and intrathoracic pressure changes caused by NSD. Besides causing hypoxia and hypercapnia via direct obstruction of the upper airway, NSD also causes hypoxia by inhibition of nasopulmonary reflex which has a positive effect on thoracic wall movement.^26^ Hypoxia in patients with NSD may cause myocardial dysfunction. Moreover, impaired quality of sleeping in NSD patients may also cause myocardial dysfunction. Sleep is not only a passive status of rest. It is a complex and dynamic process. In previous studies, one night of sleep deprivation has shown to be strongly associated with increased arterial stiffness and atrial dysfunction.^27^, ^28^ Acute sleep deprivation has some neurohormanal effects on healthy subjects, such as increased levels of thyrotropin secretion, thyroid hormons, urine catecholamines, sympathetic tone and decreased parasympathetic tone.^29^, ^30^, ^31^ Regarding LV doppler parameters which represents the myocardial functions, increased MPI, IVRT, IVCT and DT may be a consequence of these emerging neuro-endocrine changes after sleep deprivation. Small sample size was the most important limitation of our study. Another limitation was that we did not perform polysomnography on the patients before and after septoplasty in order to assess the effects of sleep deprivation for comparison with our findings.

## Conclusion

As a result, septoplasty surgery not only reduce symptoms in NSD patients but also may improve left ventricular performance. Treatment of NSD without delay is suggested to prevent possible future cardiovascular events.

## Conflicts of interest

The authors declare no conflicts of interest.
